# Correction for Wagner et al., “Draft genome sequence of potentially dikaryotic black fungus *Aureobasidium melanogenum* isolated from aircraft”

**DOI:** 10.1128/mra.00323-24

**Published:** 2024-04-22

**Authors:** Dominique N. Wagner, Vanessa A. Varaljay, Wanda J. Lyon, Audra L. Crouch, Clayton Allex-Buckner, Justin C. Biffinger, Wendy J. Crookes-Goodson, Nancy Kelley-Loughnane, Blake W. Stamps

## AUTHOR CORRECTION

Volume 13, no. 3, e00756-23, 2023, https://doi.org/10.1128/mra.00756-23. Page 1: The corresponding author should appear as shown in this correction.

